# Bioinformatic, Biochemical, and Immunological Mining of MHC Class I Restricted T Cell Epitopes for a Marburg Nucleoprotein Microparticle Vaccine

**DOI:** 10.3390/vaccines12030322

**Published:** 2024-03-18

**Authors:** Paul E. Harris, Scott Burkholz, Charles V. Herst, Reid M. Rubsamen

**Affiliations:** 1 Vagelos College of Physicians and Surgeons, Columbia University, New York, NY 10032, USA; 2 Flow Pharma Inc., Warrensville Heights, OH 44128, USA; 3 Cleveland Medical Center, University Hospitals, Cleveland, OH 44106, USA

**Keywords:** Marburg, T cell epitope, vaccine, Cynomolgus macaques, Mafa-A63

## Abstract

The Marburg virus (MARV), the virus responsible for Marburg hemorrhagic fever (MHF), is considered a top-priority pathogen for vaccine development. Recent outbreaks in Equatorial Africa have highlighted the urgency of MARV because of its high fatality rate and historical concerns about potential weaponization. Currently, there are no licensed vaccines for MARV. Existing vaccine candidates rely on attenuated recombinant vesicular stomatitis virus carrying MARV glycoprotein (VSVΔG) or the chimpanzee replication-defective adenovirus 3 vector ChAd3-MARV. Although these platforms provide significant protection in animal models, they face challenges because of their limited thermal stability and the need for cold storage during deployment in resource-poor areas. An alternative approach involves using adjuvanted poly (lactic-co-glycolic acid) (PLGA) microparticles loaded with synthetic peptides representing MHC class I—restricted T cell epitopes. This vaccine platform has demonstrated effectiveness in protecting against SARS-CoV-2 and EBoV disease in animal models and has the advantage of not requiring cold storage and remaining stable at room temperature for over six months. This report outlines the design, manufacturing, and in vivo immunogenicity testing of PLGA microparticle human vaccines designed to prevent Marburg hemorrhagic fever.

## 1. Introduction

The Centers for Disease Control Strategic Planning Workgroup has recently classified several hemorrhagic fever viruses (HFVs), including the Marburg virus, as Category A pathogens. Category A signifies that preparedness, including vaccine development, is of utmost priority [1]. This designation stems from two critical factors: the exponential significant rise in human infections originating from their zoonotic reservoirs and their potential for use as biological weapons [2].

Meadows and colleagues conducted a recent survey on zoonotic outbreaks and mortality caused by human pathogens, including those from the Coronaviridae, Filoviridae, Arenaviridae, and Paramyxoviridae families [3]. The number of outbreaks and deaths caused by these viral pathogens collectively increased exponentially from 1963 to 2019. Highlighting this unease is the recent outbreak (February 2023) of MARV hemorrhagic fever in Equatorial Guinea [4]. Of further concern is that many of these viruses (a) can be disseminated via aerosol and are infectious at low doses; (b) have no currently available or feasibly deployable vaccines [5]; and (c) have been previously researched for use as biological weapons [1,5,6,7,8].

Viral hemorrhagic fever (VHF) is a clinical syndrome marked by a sudden onset of fever and nonspecific symptoms, progressing to septic shock and often resulting in bleeding disorders [9]. Over 25 viruses from four viral families are known to cause VHFs, including Lassa, Junín, Crimean–Congo hemorrhagic fever, Rift Valley fever, yellow fever, Ebola (EBoV), and MARV. These viruses pose a significant threat because of their high rates of illness and mortality [10], coupled with a limited understanding of hemorrhagic fever syndrome [11].

The Marburg virus, the first recognized filovirus, was identified in 1967 among laboratory workers reporting symptoms of hemorrhagic fever in Marburg, Germany [12]. All infected workers had contact with materials from African green monkeys, resulting in 32 cases and an overall mortality rate of 23% [13]. In the community setting, Marburg hemorrhagic fever may spread between people via direct contact through broken skin or mucous membranes with the blood, secretions, organs, or other bodily fluids of infected people and with surfaces and materials such as bedding and clothing contaminated with these fluids [14]. Following exposure, MARV enters the body, replicates intracellularly (preferentially in monocytes, dendritic cells, and endothelial cells [15]), disseminates, and leads to a clinical syndrome comprising an Ebola-like cytokine storm [16,17,18], fever, malaise, myalgia, and blood coagulation disorders [2]. These symptoms progress to shock, multiorgan failure, and death, with a case fatality rate ranging from 20–100% [10].

Unlike Ebola VHFs [19], there are no licensed vaccines or therapeutics for Marburg virus outbreaks [20]. While MARV vaccine candidates are in clinical and preclinical testing, they rely on platforms such as attenuated recombinant vesicular stomatitis virus carrying the viral glycoprotein (VSVΔG) [21,22] or the chimpanzee replication-defective adenovirus 3 vector ChAd3-MARV [23,24]. These viral vector vaccine platforms efficiently induce robust protective antibody responses [25], but they face challenges such as cost and limited thermal stability [26,27].

Controlled-release poly (lactic-co-glycolic acid) (PLGA) microparticles loaded with synthetic peptides corresponding to immunogenic epitopes found in pathogens [28,29,30,31,32,33,34,35,36] and tumor cells [37,38,39,40] are a vaccine platform of emerging interest [41,42]. The manufacture and characterization of the PLGA microparticles used in the current study have been previously described in detail [29,30,32,36,37]. Briefly, a PLGA solution containing MHC class I and MHC class II-restricted synthetic peptide immunogens and the toll-like receptor 9 (TLR-9) oligonucleotide agonist CpG (ODN-1018) adjuvant was spray-dried to form microparticles with an average diameter of 6 μm. The microparticles were delivered in a saline-DMSO vehicle containing the toll-like receptor 4 (TLR-4) agonist monophosphoryl-lipid A (MPLA) adjuvant. In-house studies have revealed that the CpG and peptide content of the microparticles are structurally stable for more than six months when stored at room temperature, suggesting that this vaccine platform may be ideal for deployment in infrastructure-poor or conflict zones around the globe.

The design rationale for the selection of MHC class I and MHC class II-restricted peptide immunogens in this platform has focused on the following: (a) inclusion of peptide immunogens previously reported to be targets of human T cell responses, (b) toward peptides corresponding to structurally conserved, low mutational variability protein targets, and (c) inclusion of a diverse set of potential peptide immunogens capable of binding to multiple MHC molecules such that the final vaccine formulation can induce T cell responses across a wide swath of MHC molecules expressed by the world population (>85%). In preclinical testing, we found that microparticle vaccine formulations following these design guidelines conferred protection from disease to 100% of the virally challenged cohort in (a) a murine model of EBoV [32] and (b) a rhesus model of Severe acute respiratory syndrome coronavirus 2 (SARS-CoV2) [30]. Following microparticle vaccination and challenge with SAR-CoV2, macaque subjects remained pneumonia-free; however, viral titers were significant, suggesting that induction of CD8+ T cell immunity was protective but not sterilizing, unlike many of their virally delivered recombinant whole protein counterparts [43,44,45].

In this study, we discuss the development, production, and preclinical testing of a vaccine composed of adjuvanted microparticles containing immunogenic T cell epitopes derived from the MARV nucleoprotein. Recently, the Marburg proteome was screened to identify potential immunogenic epitopes that bind to human MHC molecules. However, previous reports have focused solely on bioinformatics and in silico screening studies [46,47,48,49,50,51,52,53,54,55,56,57] for human MHC molecules. Our contribution involves a bioinformatics analysis of potential immunogenic T cell epitopes specific to human MHC class I (i.e., HLA–A and B) and Cynomolgus MHC class I (Mafa MHC–A). In addition, we provide an in vitro biochemical characterization of the MHC binding strength of these peptides to their respective restriction elements. Our workflow provides a path to identifying T cell epitopes where currently available immunoinformatic tools are limited.

Despite the challenges associated with the limited availability of non-human primates (NHPs) [58], we obtained a cohort of Cynomolgus monkeys (*Macaca fascicularis*) from a colony with limited MHC diversity for in vivo/ex vivo testing of the immunogenicity of these nucleoprotein derived peptides. The peptides were delivered in vivo using the microparticle vaccine platform, and the T cell immunogenic potential of these peptides was assessed ex vivo with an interferon γ ELISpot assay. We found that the MARV nucleoprotein can be “mined” by bioinformatics and biochemical characterization of peptide binding to MHC molecules for primate immunogenic T cell epitopes, some of which may be suitable for inclusion in a thermostable MARV synthetic peptide T cell microparticle vaccine.

## 2. Materials and Methods

### 2.1. Immunoinformatics

#### 2.1.1. Protein Sequence Variability/Shannon Entropy Scores

**Figure 1:**
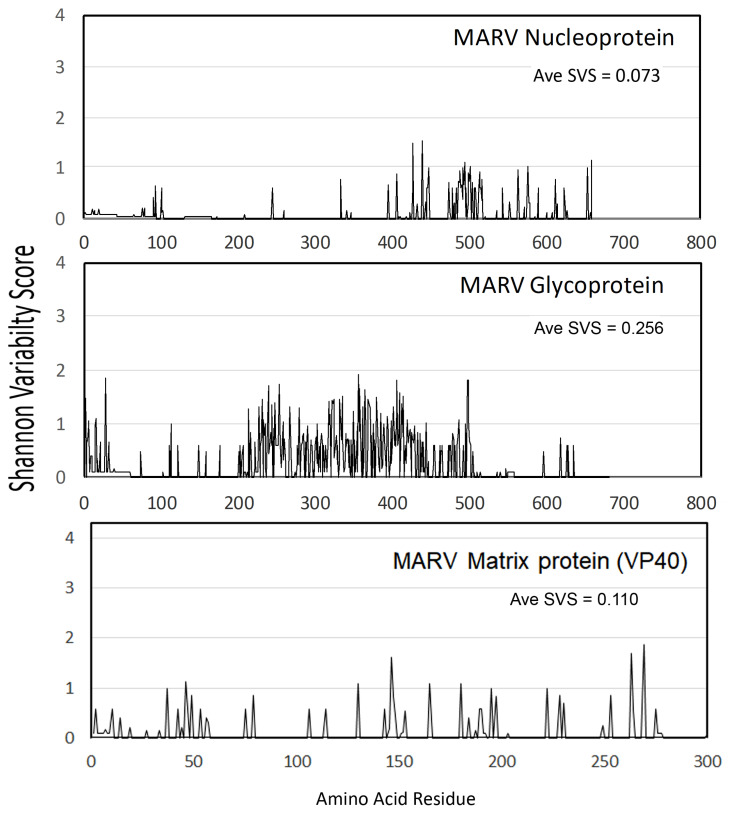
Amino acid variability analysis of MARV proteins. The sequences of MARV nucleoprotein (NP), envelope glycoprotein, and matrix protein (VP40) were downloaded from the LANL, UniProtKB, and NCBI databases, aligned, and submitted for analysis at the Protein Variability Server. The average Shannon variability score (SVS) across the entire amino acid sequence was calculated for comparison.

Multiple amino acid sequences in the fasta format of the MARV nucleoprotein (*n* = 204, 90 from Los Alamos National Laboratories (LANL), 34 from Swiss-Prot (UniProtKB), 80 from the National Center for Biotechnology Information (NCBI)), including nucleoprotein sequences obtained from sampling the zoonotic host and patients with MHF disease across both equatorial Africa as well as MARV laboratory stocks. Similar data for MARV Glycoprotein (*n* = 135) and MARV matrix protein (VP40) (*n* = 98) were also downloaded from the LANL, UniProtKB, and NCBI databases. Multiple sequence alignments for MARV nucleoprotein, envelope glycoprotein, and VP40 were obtained using the online web tool Clustal Omega (http://www.clustal.org/ accessed on 6 December 2023) and the Shannon entropy plots (where the range varies from 0 to 4.32, and the domain is the length in amino acids of the protein) in Figure 1 were calculated using the Clustal Omega alignments at the PVS server (http://imed.med.ucm.es/PVS/ accessed on 6 December 2023) [59,60].

#### 2.1.2. Antigenicity Predictions

The VaxiJen antigenicity model is a computational tool designed to predict protein and peptide antigenicity. It employs an artificial neural network that has been trained on a database containing examples of both antigens and non-antigens. Using an alignment-independent approach, VaxiJen uses characteristics such as hydrophobicity, size, charge, and aromaticity of amino acids within the primary sequence of the query protein to predict antigenicity. The predicted antigenicity of all seven MARV structural proteins was assessed using the VaxiJen v2.0 server (http://www.ddg-pharmfac.net/vaxijen/ accessed on 6 December 2023) [61]. The empirical threshold of a score of 0.4 was used to determine whether a protein or peptide might or might not be antigenic.

#### 2.1.3. In Silico MHC Class I Restricted T Cell Epitope Prediction

As the reference amino acid sequence for microparticle T cell Marburg vaccine development, we selected the ABE27012.1 nucleoprotein amino acid sequence from the laboratory MARV Angola strain [62]. Although we originally planned to study peptide immunogenicity in a rhesus NHP model, at the time of the study, no rhesus macaques were available [63,64]. Instead, we were able to secure a small cohort of Mauritian Cynomolgus macaques (*Macaca fascicularis*) (*n* = 4) sharing the Mafa-A1*063:01 or Mafa-A1*063:02 MHC class I genotype. The IEDB T cell epitope prediction server [65] allows the input of the amino acid sequence of the Mafa-A63:01 or Mafa-A63:02 MHC class I molecules (accession number AY958100.2) for use in T cell epitope predictions. The output from the Immune Epitope Database (IEDB) server, using the Marburg nucleoprotein sequence and the Mafa-A63 MHC class I sequence, is a ranked list of approximately 690 nine-mer peptides. Because suboptimal anchor residues in the peptide can cause instability of peptide-MHC-I complexes, to further limit this list, we took advantage of an additional published MHC peptide binding motif based on the elution and sequencing of peptides bound to Mafa-A63:02 MHC class I molecules [66]. The IEDB list of peptides was further prioritized by ranking the IEDB list according to the published peptide binding motif of Mafa-A63:02 MHC class I molecules, giving a higher score to nine-mer peptides having P in the P2 position and F or W in the P9 position [66]. The final list of potential MARV nucleoprotein-Mafa A63 binding peptides is given in Appendix A Table A1. To this list, we added the following: (a) three T cell epitopes peptides whose immunogenicity was known to be restricted to the Mafa-A*063:02 MHC class I gene [67,68,69], bore the above Mafa-A63 peptide binding motif, and corresponded to sequences within simian immunodeficiency virus (SIV) envelop protein or the SIV Negative Factor (NEF) transcription factor; (b) several potential MARV T cell epitopes that did not contain the above Mafa-A63 MHC class I peptide binding motif but scored high in the original IEDB predicted T cell epitope list and had possible binding affinity (<2000 nM) to human class I MHC molecules (HLA); and (c) several MARV, EBoV, HIV, or SIV nine-mer peptides that did not carry the Mafa-A63:02 MHC class I peptide binding motif and were predicted to have little to no Mafa-A63:02 binding affinity. The predicted affinity of binding to several HLA class I molecules for peptides in Appendix A Table A1 was also characterized at the IEDB server.

### 2.2. Biochemical Characterization of Peptide Binding to Mafa-A63 MHC Class I Molecules

#### 2.2.1. Peptide Synthesis, MHC Class I Peptide Binding Affinity, and Stabilization Assays

Peptides were synthesized (Peptides International, Louisville, KY, USA, InnoPep, San Diego, CA, USA and JPT Peptide Technologies GmbH, Berlin, Germany) by standard 9-fluorenylmethyloxycarbonyl (FMOC) chemistry, purified by reversed-phase high-performance liquid chromatography (>95% purity), and the correct sequence was confirmed by mass spectrometry.

The interactions of these peptides with Mafa-A63:02 MHC class I molecules were determined using a previously described biochemical binding assay [70] for measurements of peptide MHC class I binding affinity. Briefly, the Mafa-A1*063:02 MHC I allele was amplified by reverse transcription polymerase chain reaction, cloned, and sequence validated (The European Nucleotide Archive (ENA) accession AY958100.2) as previously described [70,71]. The Mafa-A1:063:02 heavy chain sequence was mutated to remove the cytosolic and transmembrane regions, and biotin acceptor sequences were added at the C-terminus of the heavy chain, as previously described [72]. The induced expression of Mafa- A1:063:02 heavy chain (HC) or beta 2 microglobulin (β2M) was performed in the Escherichia coli expression host BL21 (DE3), HC harvested as inclusion bodies, extracted into a urea buffer, purified by gel filtration, and biotinylated as previously described [73]. Next, measurements of MHC class I peptide complex binding affinity [70] were performed in a refolding assay. Purified urea-denatured Mafa-A63 heavy chains were diluted 100-fold into a buffer containing β2M and varying concentrations (5 × 10^−3^ to 5 × 10^4^ nM) of the test peptide. Each peptide was assayed at least two to three times in the refolding assay. Complexes were allowed to form for 24 h at 18 °C and then captured on a W632 mAb-coated enzyme-linked immunosorbent assay (ELISA) plate, washed, and captured complexes were detected with a polyclonal anti β2M—horseradish peroxidase (HRP) conjugated antibody. Once developed, the colorimetric reaction was read at 450 nm (OD450) using an ELISA plate reader. The OD450 data versus peptide concentration data were fitted to a four-parameter logistic (4PL) nonlinear regression model using a web-based tool (https://mycurvefit.com/ accessed on 6 December 2023), and the peptide concentration at half-maximal folding, equivalent to the Kd for peptide-Mafa-A63 MHC class I binding, was extracted from the estimated regression equation. The estimated Kd measurements are the mean of two to three refolding assays. The MHC class I peptide stability assay was performed as previously reported [74] with modifications. Refolded test peptide-Mafa A63 MHC class I—β2M complexes were captured via their biotin tag on ELISA plates, incubated in 0, 2, 4, and 6 M urea for 2 h at 18 °C, and then detected with W632 as described above. The stability challenge outcome measure, rather than the half-life of peptide-Mafa A63 MHC class I complexes incubated at 37 °C, was calculated as the average yield (OD450) of stable peptide-Mafa A63 MHC class I—β2M complexes incubated in the presence of four concentrations of the urea chaotrope minus the associated background relative to the stability of the reference Mafa-A063 binding SIV-NEF peptide, RPKVPLRTM, and expressed as a percentage.

The affinity binding data (as OD450) for all peptides was then percentile ranked, and the stability of the MHC complexes for each peptide, expressed as a percent, was combined in a data frame (66 × 9 elements) and submitted to Morpheus (https://software.broadinstitute.org/morpheus accessed on 6 December 2023) for dimension reduction by t-distributed stochastic neighbor embedding (t-SNE) and ranked by the t-SNE T2 axis for grouping of peptides by similarity of binding to Mafa-A063 MHC Class I binding.

From the list of peptides in Appendix A Table A1, we selected 16 nine-mer, 1 eight-mer, and 3 ten-mer peptides with IEDB-predicted HLA binding affinity < 600 nM (Appendix A Table A2). The binding affinity of each of these peptides to HLA class I molecules was measured in a classical competition assay. The set of 29 HLA class I molecules used in this assay is given in Appendix A Table A3. Each peptide was tested for its capacity to bind to the corresponding predicted HLA class I allele(s). Additional molecules of HLA alleles of interest were selected for binding studies with each candidate peptide if the predicted binding affinity was <2000 nM. The purification of MHC molecules for binding studies by affinity chromatography and the competition assay was performed as detailed elsewhere [75]. In brief, varying concentrations of candidate test peptide and 0.1–1 nM of a radiolabeled reference peptide were co-incubated at room temperature or 37 °C with purified HLA molecules in the presence of a cocktail of protease inhibitors. Following a 2- to 4-day incubation, HLA molecule-bound radioactivity was determined by capturing MHC/peptide complexes on mAb W6/32- or B123.2-coated Lumitrac 600 plates (Greiner Bio-one, Frickenhausen, Germany) and measuring bound cpm using the TopCount (Packard Instrument Co., Meriden, CT, USA) microscintillation counter. The concentration of peptide yielding 50% inhibition of binding of the radiolabeled peptide was calculated. Under the conditions utilized, where [label] < [MHC] and IC50 ≥ [MHC], the measured IC50 values are reasonable approximations of true Kd. Each competitor peptide was tested at six different concentrations covering a 100,000-fold range in three or more independent experiments. As a positive control, the unlabeled version of the radiolabeled probe was also tested in each experiment.

#### 2.2.2. Preparation of Adjuvanted MARV Microspheres for In Vivo Studies

**Figure 2:**
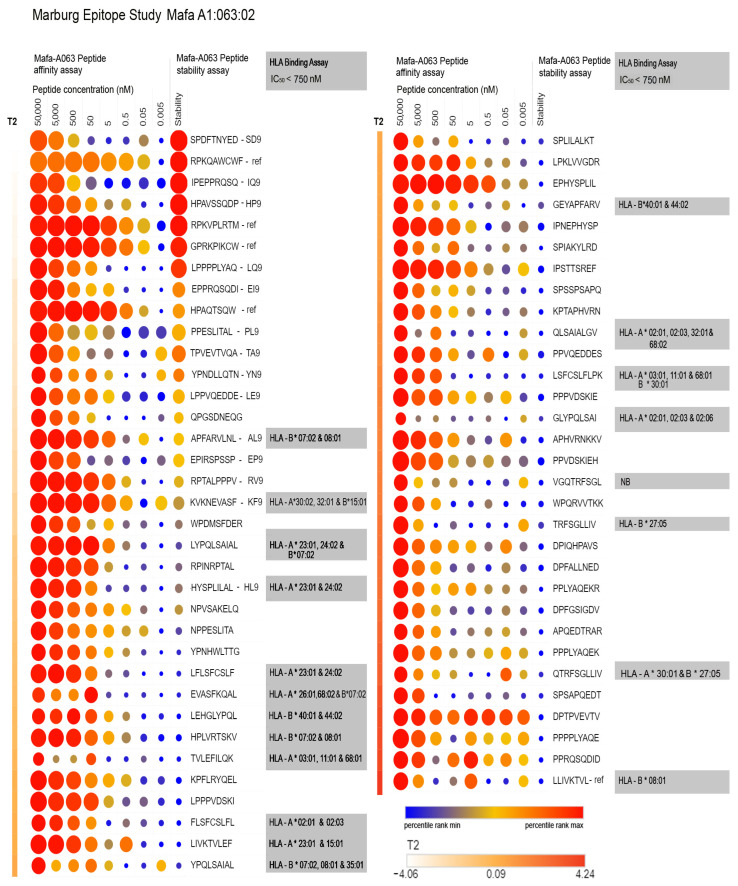
t-SNE–based ranking of MARV test peptides based on Mafa-A63 binding affinity and stability. Peptides from Appendix A Table A1, including reference peptides, were characterized in Mafa-A63 binding affinity and stability studies. The entire binding affinity data set for 66 peptides was percentile-ranked. The stability assay outcome measure was separately ranked as the percentile score. The binding and stability data as percent were analyzed by t-distributed stochastic neighbor embedding (t-SNE) and ranked by the second dimension of the t-SNE plot (T2) (Orange gradient line at left). Both binding affinity and stability data are illustrated above in a heat map, where the magnitude of the percentile rank is reflected in both the size and color of the associated measurement. As expected, the three reference positive control peptides (RPKVPLRTM, GPRKPIKCW, HPAQTSQW), with known affinity and stability of Mafa-A063 binding, scored high on the t-SNE T2 axis. Peptides from Appendix A Table A1 were submitted to IEDB, and their predicted HLA class I molecule affinity was calculated and ranked. Twenty peptides with predicted high affinity binding to HLA class I molecules were assayed using a soluble HLA class I molecule-based competitive binding assay.

The selection of peptides representing potential MARV T cell epitopes was based on the Mafa-A063 binding affinity and stability (Appendix A Table A2), with priority given to peptide sequences carrying the published peptide binding motif of Mafa-A063 MHC class I molecules [66] and the highest measured affinity/stability. As a second criterion, we selected two additional peptides that demonstrated high binding activity to human HLA class I molecules (Figure 2). The amino acid sequences of the MARV peptides selected for in vivo testing are given in Appendix A Table A4. Additional peptides could not be selected because of our limitations in the total number of peptides that could be tested in vivo.

The peptide epitopes used in this study were delivered in vivo by intramuscular injection of a formulation of PLGA microspheres containing the corresponding synthetic nine-mer peptides and the TLR-9 agonist CpG oligonucleotide adjuvant in a vehicle containing the TLR-4 agonist monophosphoryl lipid A (MPLA). The rationale for choosing the delivery platform and the basic manufacturing scheme used in production has been previously described [29,30,32,36,37]. In brief, room-temperature solutions of synthetic peptide (Appendix A Table A4) and CpG oligonucleotide were mixed with a solution of PLGA in acetone. A second batch of microspheres was prepared as above but with the addition of two additional peptides, ILMQYIKANSKFIGIPMGLPQSIALSSLMVAQ (TpD) [76] and QYIKANSKFIGITEL (TT830-844) [77], which are reported to be promiscuous MHC class II-binding T helper (Th) epitopes in Cynomolgus macaques.

The formulation was then processed using a precision spray-drying device (GEA, Columbia, MD, USA) and passed through a nitrogen gas-filled drying chamber at 65 °C to allow evaporation of the acetone. The dry microsphere stream was analyzed in real-time using a laser particle size analyzer (SprayTech, Malvern Instruments, Malvern, PA, USA) before collection (Buchi cyclone dryer) as a dry powder. The microsphere powder was admixed with mannose and hypromellose as resuspension aids. At the time of delivery, a diluent containing oleic acid, 2% dimethylsulfoxide (DMSO), and MPLA (20 μg/mL) in an aqueous buffer was used to reconstitute the microsphere formulation. Each microsphere contained peptide loaded at approximately 0.1% by weight and CpG 0.01% by weight. Monitoring the microsphere diameters allowed the production of microspheres with a mean diameter of 10 ± 2 microns. This diameter was selected for formulation to ensure delivery to antigen presenting cells (APC) via phagocytosis of no more than 1–4 microspheres per cell, which have average diameters of 13 microns. GMP manufacturing protocols were employed using GMP grade synthetic peptides (Peptides International, Louisville, Kentucky, USA, InnoPep, San Diego, CA, USA and JPT Peptide Technologies GmbH, Berlin, Germany), GMP grade CpG oligodeoxynucleotides (Trilink Biosciences, San Diego, CA, USA), and GMP grade MPLA (Avanti Polar Lipids, Alabaster, AL, USA). The CpG oligonucleotide and MPLA used in this study were manufactured using the same chemical compositions as the equivalent materials used in FDA-approved vaccines. Assessment of the thermal stability of the synthetic peptides within the microspheres has been previously reported [36]. The peptide content and structure in microspheres were determined by high-performance liquid chromatography after two months of room-temperature storage. We found that over 99% of the peptide was structurally intact.

### 2.3. In Vivo Experiments

Mauritian Cynomolgus macaques were selected for testing because of their well-characterized MHC immunogenetics [78]. Macaques were obtained from the testing facility holding pool of Worldwide Primates (Miami, FL, USA). Before shipping, peripheral blood samples were obtained from a cohort of 26 macaques and shipped to the Wisconsin National Primate Research Center for MHC class I and class II typing as previously described [79]. From the MHC Class I typing data, we selected four macaques (3 males/1 female, 3–4 years old, and 2–6 kg) sharing the MHC class I genotype Mafa-A1*063:01 or Mafa-A1*063:02 genotype. In vivo testing of MARV microsphere immunogenicity was performed at AmplifyBio (West Jefferson, OH, USA).

#### 2.3.1. Animal Care and Housing

General procedures for animal care and housing met AAALAC International recommendations (AAALAC unit # 000446), current requirements stated in the “Guide for the Care and Use of Laboratory Animals” (National Research Council, Current Edition), and current requirements as stated by the US. Department of Agriculture through the Animal Welfare Act, as amended, and conformed to the applicable testing facility SOP. Prior to study initiation, the study design was reviewed and approved by the Institutional Animal Care and Use Committee (IACUC).

Animals were socially housed during the study. The temperature and humidity ranges of the study room were set to maintain 74 ± 10 °F and 50 ± 20%, respectively. The light cycle was set to maintain 12 h on/12 h off. Animals were provided with fresh water ad libitum and PMI Certified Primate Diet (LabDiet 5048) twice daily, except during specified fasting periods or when the animal was away from its home cage for study events (e.g., when placed in restraint chairs for dose administrations and blood collections). The diet was also supplemented with fresh fruits, vegetables, or other supplemental enrichment (e.g., manipulatives). Animals were fasted, as appropriate, for sedation.

#### 2.3.2. MARV Microsphere Immunization

Macaques were vaccinated according to the schedule shown in Table 1. Peripheral blood sampling was scheduled before vaccination but under the same session of intramuscular (IM) Ketamine-induced sedation (5–20 mg/kg). Microsphere vaccine formulations were suspended in the diluent to deliver 20 mg/mL of microspheres. Sedated macaques received IM injections of vaccine to both right and left (1 mL each) thigh muscles and one arm (1 mL). Macaques received four weekly injections of MARV vaccine microspheres, followed by three additional doses of MARV microspheres supplemented with the TpD and TT_830–844_ peptides, on days 84, 110, and 150. Between MARV microsphere dosing and the last MARV boost doses, macaques received four IM doses (0.5 mL at 2.5 mg/0.5 mL) on (days 63, 81, 110, and 150) of a human use approved Diphtheria and tetanus toxoids and Acellular Pertussis vaccine (TDaP, Adacel, Sanofi-Pasteur, Inc., Bridgewater, NJ, USA).

#### 2.3.3. ELISpot Assays

Femoral vein peripheral blood (4–6 mL) was collected from each animal into a BD Vacutainer^®^ K2EDTA tube (Becton, Dickinson and Company, Franklin Lakes, NJ, USA), diluted 1:2 with Hanks buffered salt solution (HBSS), and processed by buoyant density centrifugation using Lymphoprep density gradient medium diluted 9 parts medium plus 1 part HBSS to obtain peripheral blood mononuclear cells (PBMCs). Collected macaque PBMCs were washed in HBSS and diluted to 2.5 × 10^6^ cells/mL in complete growth medium (CGM) (i.e., Gibco RPMI 1640 high glucose medium, HEPES, Glutamax, supplemented with 10% fetal bovine serum, non-essential amino acids, sodium pyruvate, and antibiotics) and then assessed for MARV peptide immunoreactivity using an ELISpot assay. In brief, ELISpot assay plates (MabTech Inc., Cincinnati, OH, USA) specific for the detection of primate IFN γ were used according to the manufacturer’s instructions. Diluted PBMC cells were dispensed (100 µL/well) into a 96-well plate that had been pre-plated using an automated liquid handler (Opentrons, Queens, NY, USA) under sterile filtered air flow. One hundred µL of CGM alone (negative control), Phytohemagglutinin-A/L (PHA-L) in CGM at 5 µg/mL (positive control), MARV peptides (Appendix A Table A4) at 10–20 g/mL, tetanus toxoid (positive control) (List laboratories, Campbell, CA, USA) at 2 g/mL, or tetanus peptide (positive control) (TT_830–844_) at 10 g/mL were added. Peptides used for macaque microsphere immunization were added to the wells at a concentration of 10 g/mL. All samples were assayed in duplicate. Plates were incubated at 37 °C/5% CO_2_ for 36–48 h, after which plates were thoroughly washed. The conjugated detection antibody was then added and incubated, followed by additional washing. Wells were developed using 3,3′,5,5′-tetramethylbenzidine as the substrate. Counts were performed at the Cellular Technology Limited Corporation (Shaker Heights, OH, USA) using an S6 Entry M2 immunospot analyzer (CTL, Shaker Heights, OH, USA), and all well images were quality-controlled on-site. All spot-forming cell counts reported are the result of averaging counts from the duplicate wells. PBMC-peptide immunoreactivity was considered to be positive if the average number of spot-forming cells (SPC) per million plated PBMC of the duplicate wells was greater than or equal to two times the average of its corresponding control well with CGM but no peptide. A one-sided t-test for the duplicate wells was <0.05 relative to all the control wells (complete growth medium alone wells, *n* = 8) on the ELISpot plate.

## 3. Results and Discussion

### 3.1. Immunoinformatic Screening of Marburg Proteins: Shannon Sequence Variability and Antigenicity Predictions

The calculated Shannon sequence variability scores for proteins are well correlated with structural entropy and are considered a metric of the compositional stability and packing density of proteins in solution. Protein domains with lower packing density and higher local flexibility have increased Shannon variability scores and are associated with higher mutation and structural variability [80,81]. Conversely, low sequence variability in a protein region suggests that this region is essential for protein function, making it less likely to mutate without compromising the fitness of the virus [82]. The Shannon variability or entropy metric has been frequently used in the design of vaccines for HIV [83] and SARS-CoV-2 [30].

We analyzed the Shannon entropy scores for different proteins of MARV, specifically the nucleoprotein, glycoprotein, and matrix proteins. We calculated the average Shannon sequence variability score across the entire length of each protein (Figure 1). Our findings showed that the MARV nucleoprotein amino acid sequences, as sourced from databases, exhibited lower sequence variability than the MARV matrix protein and envelope glycoprotein.

As a second-level triage, we examined the predicted antigenicity of the seven MARV structural proteins (Table 2). While all the MARV proteins scored as possibly antigenic (score > 0.4), based on the low mutational probability predicted by the Shannon sequence variability score and our previous successful experiences with the SARS-CoV2 and EBoV nucleoprotein as the viral target molecule [30,32], we decided to focus on identifying potential T cell epitopes within the MARV nucleoprotein.

### 3.2. Immunoinformatic Screening of Marburg Proteins: Prediction of Mafa–A063 T Cell Epitopes

The output from the IEDB server, using the Marburg nucleoprotein sequence and the Mafa-A063 MHC class I sequence as input, is a ranked list of approximately 690 nine-mer peptides. These potential T cell epitopes were next ranked by the published peptide binding motif of Mafa-A63:02 MHC class I molecules, giving a higher score to nine-mer peptides having P in the P2 position and F or W in the P9 position [66]. To this list, we added both positive and negative control peptides to aid in the physical characterization of the binding of these peptides to both Mafa–A063 molecules and several alleles of HLA class I molecules to obtain a set of 66 peptides (Appendix A Table A1).

### 3.3. MARV Peptide Binding to Mafa-A063 and HLA Class I Molecules

The in vitro binding and stability of the 66 peptides to Mafa-A063 MHC class I molecules are summarized in Figure 2. We observed that both MARV test peptides and reference peptides, carrying the expected peptide binding motif of Mafa-A063, generally showed the highest binding affinity and stability relative to other MARV peptides lacking the P2-proline pattern. We also observed several moderate-to-high-affinity binding peptides to human HLA class I molecules, three of which shared the Mafa-A063 peptide-binding motif. We ranked the test peptides using a combination of both measured binding affinity and stability to obtain a set of peptides that would be most suitable for in vivo immunogenicity testing based on a set of three reference peptides whose immunogenicity was known to be restricted to the Mafa-A*063:02 MHC class I gene [67,68,69]. These synthetic peptides (Appendix A Table A2) were incorporated into adjuvanted PLGA microspheres and tested in vivo for their ability to evoke a T cell response in the Cynomolgus primate model. 

### 3.4. In Vivo Immunogenicity Testing of MARV Peptide-Containing Microspheres in Cynomolgus Macaques Carrying the Mafa–A1*063 MHC Class I Allele

**Figure 3:**
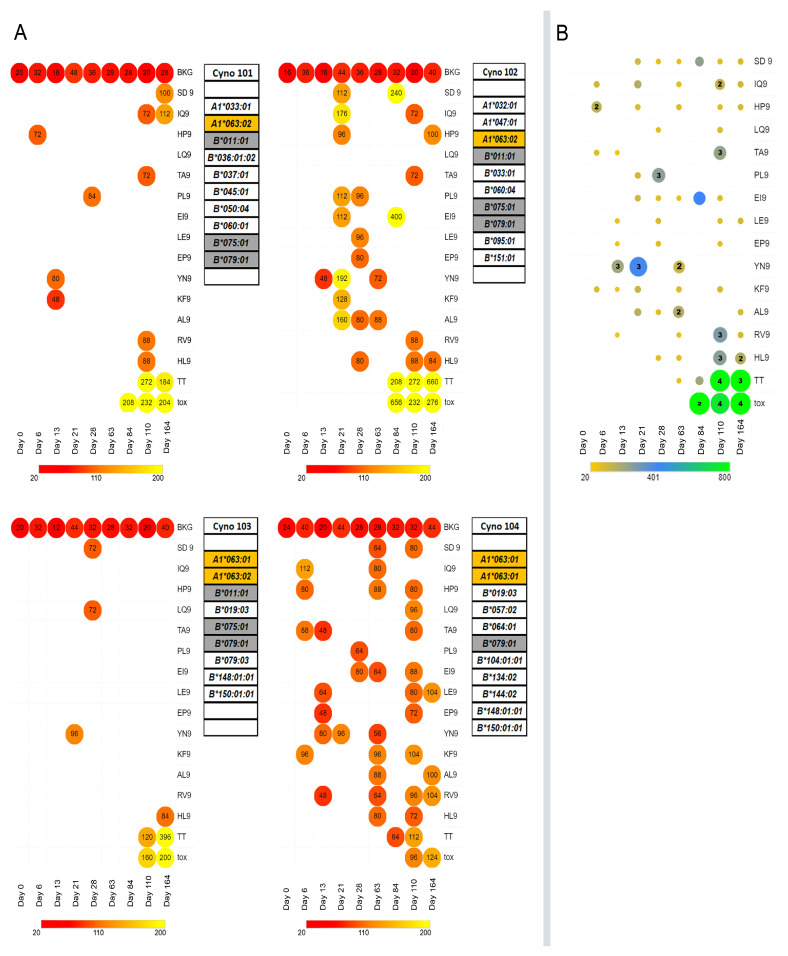
Time course study of macaque ELISpot reactivity to selected MARV peptides following microsphere vaccination. (**A**) Four Cynomolgus macaques carrying the Mafa-A*062 MHC genotype were vaccinated with synthetic peptides corresponding to Mafa-A063 restricted CD8+ T cell epitopes. On the day indicated on the *x*-axis, PBMCs were harvested from the macaques and processed for ELISpot analysis. PBMCs (2.5 × 10^5^/well) and the indicated synthetic peptide were added to duplicate wells. PBMCs were also added to ELISpot plate wells with complete growth medium (CGM) with no peptide (BKG—8 wells/plate), ELISpot plate wells with CGM and tetanus toxoid (tox), and ELISpot plate wells with CGM and tetanus toxoid peptide TT830–844. Following incubation, the plates were developed for gamma interferon immunoreactivity according to the manufacturer’s instructions. The developed plates were machine counted using an S6 Entry M2 immunospot analyzer. The averages of duplicate wells were compared with plate background wells using Student’s t-statistics. Only wells that showed (1) average responses greater than 2X the background and (2) statistically significant (one-sided, *p* < 0.05) responses greater than the plate background were considered positive. Heat maps of the responses are summarized in the figure above. The magnitude of each response, expressed as the number of Interferon γ spot-forming cells (SFC) per million PBMCs from the animals included in the study, is given inside the circle. (**B**) The magnitude of the total number of Interferon γ spot-forming cells, summed across all four study macaques, is shown in a heat map format (yellow to blue to green). The number of macaques responding to the same peptide on the same day is shown within the circle when greater than 1.

Four macaques were administered four weekly IM injections of MARV peptide-containing microspheres (100 mg microsphere formulation total dose/week), and the T cell response was studied using ELISpot (Figure 3, Panel A). Peripheral T cell immunoreactivity to MARV peptides was detected in all four macaques, but the pattern and range of immunoreactivity toward the challenge peptide varied widely between macaques. Macaque ELISpot signal strengths as SFC/million cells were similar to those of previous single peptide studies in primates [84]; however, we did observe that some ELISpot responses appeared, then disappeared, only to appear again during the study. The waxing and waning of T cell IFN γ responses during longitudinal studies of T cell reactivity has been previously reported [85,86,87] and is consistent with T effector cell differentiation and migration from the peripheral circulation into the tissue to become resident T memory cells [88].

Unlike most humans, cynomolgus macaques do not receive TDaP vaccination as part of their routine veterinary care. To induce an anti-tetanus toxoid Th cell response, macaques were administered four doses of TDaP vaccine (the only NHP-approved vaccine containing tetanus toxoid) starting on day 63. Microspheres containing MARV peptides and tetanus toxoid T helper cell epitopes (TpD and TT_830–844_) were used in the boost phase of vaccination that started on day 84 of the experiment. We reasoned that the inclusion of Th epitopes in the MARV vaccine microspheres might enhance the CD8+ T cell responses to the MARV T cell epitopes presented by APC through the provision of CD4+T cell help. Although vaccination with tetanus toxoid, either as whole protein or long synthetic peptides, did elicit an anti-tetanus T cell IFN γ response as visualized by the ELISpot assay (tox and TT, Figure 3), it is not clear from our study whether the provision of Th epitopes in the microsphere vaccine had an effect on the in vivo anti-MARV peptide MHC class I restricted T cell response.

One workaround to the apparent lack of MHC class II-restricted T helper activity provided by the TpD and TT (830–844) peptides would be to substitute them with MHC class II T helper epitopes from the Marburg proteins themselves (e.g., MARV glycoprotein) as suggested by the protective efficacy of viral vector vaccines delivering both MARV nucleoprotein and glycoprotein [89]. Current immunoinformatic tools, however, are not tailored to the prediction of peptide binding affinities for mafa-MHC Class II molecules, and epitope predictions must be based on the homologies of the mafa MHC class II molecules to their human or rhesus counterparts. Examples of these “best guess” mafa-MHC class II binding epitopes are listed in Appendix A Table A4.

The set of peptides selected for in vivo testing were selected for high affinity and stable binding to Mafa-A063 MHC class I molecules because efficient MHC class I binding is a necessary but not sufficient condition of immunogenicity [74,90]. All the selected MARV peptides tested were found to evoke MARV peptide-specific peripheral T cell IFNγ secretory responses, consistent with previous reports [91], but in disagreement with the predicted immunogenicity of the set of peptides predicted by the VaxiJen model (Appendix A Table A5). This discordance may be attributable to insufficient training of the model on immunogenic epitopes in the Cynomolgus model. We further note that despite the genetic similarities at the MHC Class I locus of the macaque study cohort, the pattern of T cell responses between probands was dissimilar, probably attributable to other genetic influences (e.g., genes associated with antigen processing and presentation or MHC Class I expression or T Cell receptors) lying both within and outside the MHC.

Several reverse vaccinology or immunoinformatic (in silico only) studies of Marburg structural proteins have been recently published [46,47,48,49,50,51,52,53,54,55,56,57]. The bulk of these studies limited their characterization of potential T cell epitopes to the envelope glycoproteins, VP 24 matrix protein, VP30 transcription factor, VP35 polymerase cofactor, and VP matrix protein of MARV [46,51,56,57]. However, two studies reported on potential HLA class I or class II-restricted T cell epitopes within the MARV nucleoprotein [49,55]. Sami et al. [55] reported on two potentially HLA-B*08:01 restricted T cell epitopes, one of which is common to our list of peptides studied for binding to Mafa-A063 molecules (high-affinity binding). Baral et al. [49] also characterized potential MARV nucleoprotein-derived HLA Class I restricted T cell epitopes. They identified 12 potential HLA class I restricted T cell epitopes based on predicted HLA binding by the IEDB server, of which four peptides overlapped with the potential T cell epitopes characterized in our study (Appendix A Table A1). Three of these peptides, YPQLSAIAL, GLYPQLSAI, and LEHGLYPQL, showed high affinity binding to soluble HLA molecules in our studies (Appendix A Table A2).

## 4. Conclusions

Systemic vaccination of Cynomolgus Macaques with a Venezuelan equine encephalitis virus replicon carrying MARV nucleoprotein has demonstrated only partial success in providing protection against MARV challenge [89]. Our main goal has been to develop a vaccine to protect against Marburg hemorrhagic fever disease that offers unique advantages (detailed in Appendix A Table A6) not available in existing Marburg vaccines currently being tested in clinical trials [92,93]. These advantages included (a) the ability to distribute the vaccine without the need for refrigeration, removing the dependency on cold storage logistics; (b) the absence of animal products in its composition, making it compliant with halal standards [94]; and (c) being deliverable to the mucosa and potentially suitable for multiple dosing via an inhalation route [95,96]. This report represents an intermediate step, a preclinical study in a NHP model, towards that objective.

We characterized the in vivo immunogenicity of a set of potential T cell epitopes within the MARV nucleoprotein, predominantly carrying the Mafa-A63 peptide binding motif (i.e., proline in the P2 position and, to a lesser extent, phenylalanine, tryptophan, or leucine in the P9 position). The Mafa-A63 peptide binding motif [66] and the HLA-B07:02 peptide binding motif [97] are very similar at the P2 and P9 positions. One peptide (AL9) carrying this motif was found to be immunogenic in the context of Mafa-A063 (Figure 2) and demonstrated in vitro binding activity to HLA-B07:02 molecules, as was predicted in silico. There were several other potential MARV T cell epitopes that were demonstrated to bind to HLA-B07:02 as well as other HLA class I molecules, but they were not studied in vitro because of poor Mafa-A063 class I molecule—peptide complex stability.

The provision of tetanus toxoid-based MHC class II-restricted peptide epitopes in the microsphere vaccine did not clearly enhance the mafa-MHC class I-restricted T cell responses to the MARV nucleoprotein peptide epitopes. In future iterations of the microsphere vaccine, substitution of the tetanus peptide epitopes with MHC Class II binding epitopes from other MARV proteins (e.g., envelop glycoprotein) is indicated. The value of these potential MARV MHC class I and class II-restricted T cell epitopes toward the construction of a human T cell epitope vaccine for protection against Marburg virus infection awaits further validation.

## Figures and Tables

**Table 1:** Vaccination and blood draw schedule.

Experiment Day	Blood Draw and ELISpot	MARV Microsphere Vaccination	TDaP Vaccination	MARV Microsphere with TpD and TT_830–844_ Vaccination
Day 0 (Baseline)	X	X		
Day 6	X	X		
Day 13	X	X		
Day 21	X	X		
Day 28	X			
Day 63	X		X	
Day 81	No ELISpot		X	
Day 84	X			X
Day 110	X		X	X
Day 150	No ELISpot		X	X
Day 164	X			

**Table 2:** VaxiJen predicted antigenicity scores.

MARV Protein Name	Accession Number	VaxiJen Overall Protective Antigen Prediction Score
VP30	ABA87128.1	0.5636
glycoprotein GP	CAA78117.1	0.5481
VP24	ABA87129.1	0.5423
Nucleoprotein (NP)	ABE27012.1	0.4784
Polymerase (L)	ABA87130.1	0.4428
VP35	ABA87125.1	0.4316
matrix protein VP40	ABA87126.1	0.4107

## Data Availability

The data presented in this study are available on request from the corresponding author.

## Appendix A

**Table A1:** Synthetic peptide sequences used in binding and stability assays.

Number	Peptide Sequence	IEDB Focused on the Mafa-A63 Peptide Binding Motif	IEDB High Binding Score Rank without Motif	Mafa-A063 Positive or Negative Control Peptide
1	APFARVLNL	X		
2	APHVRNKKV	X		
3	APQEDTRAR	X		
4	DPFALLNED	X		
5	DPFGSIGDV	X		
6	DPIQHPAVS	X		
7	DPTPVEVTV	X		
8	EPHYSPLIL	X		
9	EPIRSPSSP	X		
10	EPPRQSQDI	X		
11	HPAVSSQDP	X		
12	HPLVRTSKV	X		
13	IPEPPRQSQ	X		
14	IPNEPHYSP	X		
15	IPSTTSREF	X		
16	KPFLRYQEL	X		
17	KPTAPHVRN	X		
18	LPKLVVGDR	X		
19	LPPPPLYAQ	X		
20	LPPPVDSKI	X		
21	LPPVQEDDE	X		
22	NPPESLITA	X		
23	NPVSAKELQ	X		
24	PPESLITAL	X		
25	PPLYAQEKR	X		
26	PPPLYAQEK	X		
27	PPPPLYAQE	X		
28	PPPVDSKIE	X		
29	PPRQSQDID	X		
30	PPVDSKIEH	X		
31	PPVQEDDES	X		
32	QPGSDNEQG	X		
33	RPINRPTAL	X		
34	RPTALPPPV	X		
35	SPDFTNYED	X		
36	SPIAKYLRD	X		
37	SPLILALKT	X		
38	SPSAPQEDT	X		
39	SPSSPSAPQ	X		
40	TPVEVTVQA	X		
41	WPDMSFDER	X		
42	WPQRVVTKK	X		
43	YPNDLLQTN	X		
44	YPNHWLTTG	X		
45	YPQLSAIAL	X		
46	EVASFKQAL		X	
47	GEYAPFARV		X	
48	GLYPQLSAI		X	
49	HYSPLILAL		X	
50	KVKNEVASF		X	
51	LEHGLYPQL		X	
52	LIVKTVLEF		X	
53	QLSAIALGV		X	
54	TRFSGLLIV		X	
55	TVLEFILQK		X	
56	VGQTRFSGL		X	
57	RPKQAWCWF			Control HIV ENV peptide with a motif
59	FLSFCSLFL			Negative Control-Low IEDB rank
60	LFLSFCSLF			Negative Control-Low IEDB rank
61	LYPQLSAIAL			Negative Control-Low IEDB rank
62	QTRFSGLLIV			Negative Control-Low IEDB rank
63	LSFCSLFLPK			Negative Control-Low IEDB rank Control
64	**LLIVKTVL**			Negative control reference: EBOV NP (8 mer)
58	**RPKVPLRTM**			Positive control reference SIV NEF [69]
65	**GPRKPIKCW**			Positive control reference–SIV GP [67]
66	**HPAQTSQW**			Positive control reference: SIV NEF [68]

Notes: HIV ENV, Human immunodeficiency virus envelope protein, EBOV NP, Ebola virus nucleoprotein, SIV GP, Simian immunodeficiency virus glycoprotein, SIV NEF Simian immunodeficiency virus-negative regulatory factor.

**Table A2:** Synthetic peptides incorporated into PLGA microspheres.

Number	Peptide Sequence	IEDB-Predicted Binding Allele and Affinity (nM)	Measured Affinity as IC_50_ (nM)	IEDB-Predicted Binding Allele and Affinity (nM)	Measured Affinity as IC_50_ (nM)	IEDB-Predicted Binding Allele and Affinity (nM)	Measured Affinity as IC_50_ (nM)
1	APFARVLNL	HLA-B*07:02–32	**0.21**	HLA-B*08:01–287	**67**		
2	HPLVRTSKV	HLA-B*07:02–87		HLA-B*08:01–268			
3	YPQLSAIAL	HLA-B*07:02–15	**44**	HLA-B*08:01–481	**17**		
4	EVASFKQAL	HLA-A*26:01–574	**86**	HLA-A*68:02 ^1^	**2.7**	HLA-B*07:02	**250**
5	GEYAPFARV	HLA-B*40:01–145	**107**	HLA-B*44:02	**214**		
6	GLYPQLSAI	HLA-A*02:01–18	**5.9**	HLA-A*02:03	**1.4**	HLA-A*02:06	**6.9**
7	HYSPLILAL	HLA-A*24:02–190	**64**	HLA-B*23:01	**3.5**		
8	KVKNEVASF	HLA-B*15:01–32	**5.6**	HLA-A*32:01	**2.9**	HLA-A*30:02	**158**
9	LEHGLYPQL	HLA-B*40:01–23	**14**	HLA-B*44:02	**3.4**		
10	LIVKTVLEF	HLA-B*15:01–26	**0.80**	HLA-A*23:01	**109**		
11	QLSAIALGV	HLA-A*02:01–28	**3.9**	HLA-A*02:03 HLA-A*02:06	**4.4** **12**	HLA-A*32:01 HLA-A*68:02	**81** **11**
12	TRFSGLLIV	HLA-B*27:05–116		HLA-B*39:015–344			
13	TVLEFILQK	HLA-A*03:01–64	**19**	HLA-A*11:01 HLA-A*68:01	**2.2**	HLA-A*30:01	**365**
14	VGQTRFSGL	HLA-B*08:01–442					
15	FLSFCSLFL	HLA-A*02:01–4	**1.8**	HLA-A*02:03	**29**	HLA-A*02:06	**121**
16	LFLSFCSLF	HLA-A*24:02–98	**633**	HLA-A*23:01	**33**		
17	LYPQLSAIAL	HLA-A*24:02 −148	**179**	HLA-B*07:02–120	**134**	HLA-A*24:02	**179**
18	QTRFSGLLIV	HLA-B*27:05–600	**533**	HLA-A*30:01	**421**	HLA-B*15:01	**4682**
19	LSFCSLFLPK	HLA-A*03:01–38	**4.3**	HLA-A*11:01 HLA-A*30:01	**0.68** **238**	HLA-A*31:01 HLA-A*68:01	**686** **1.0**
20	LLIVKTVL	HLA-B*08:01–179	**6.1**				

Notes: Where no predicted affinity measurements are given, the result to the right is the actual measured affinity as IC50 from the competition assay.

**Table A3:** Mafa MHC class I binding peptides tested for binding to HLA Class I molecules.

Peptides from Appendix A Table A2 Tested against the HLA Allele	Peptides Tested	# of. Binders
A*01:01	1	0
A*02:01	5	3
A*02:03	7	3
A*02:06	9	3
A*03:01	2	2
A*11:01	2	2
A*23:01	4	4
A*24:02	3	2
A*26:01	3	1
A*30:01	4	3
A*30:02	1	1
A*31:01	2	0
A*32:01	4	3
A*33:01	3	0
A*68:01	3	2
A*68:02	5	2
B*07:02	7	5
B*08:01	7	4
B*15:01	7	3
B*27:05	2	1
B*35:01	8	1
B*39:01	0	0
B*40:01	2	2
B*44:02	2	2
B*44:03	2	0
B*51:01	5	0
B*53:01	5	0
B*57:01	1	0
B*58:01	3	1

**Table A4:** Potential Mauritian Cynomolgus macaque MHC class II binding T helper epitopes from MARV envelope glycoprotein (GP).

Peptide Sequence	ID	DRB1_0104 ^1^		DRB1_0901		DRB1_1602		DRB5_0101	
		Core	nM	Core	nM	Core	nM	Core	nM
QYIKANSKFIGITEL	Tet 830–844	YIKANSKFI	27	YIKANSKFI	13	YIKANSKFI	13	YIKANSKFI	17
VAD**SPLEASKRW**AFRTGVPPKNVEYTE ^2,3^	GP 59–85	FRTGVPPKN	122	FRTGVPPKN LEASKRWAF	85	WAFRTGVPP	165	FRTGVPPKN	46
FISLILIQGIKTLPILEIASNNQPQN	GP 7–32	IKTLPILEI	19	IKTLPILEI IQGIKTLPI	62	IKTLPILEI	55	IQGIKTLPI ILEIASNNQ	117
RVFTEGN**IAAMIVNKT**VHKMIFSRQ ^4^	GP 158–182	IAAMIVNKT	69	IAAMIVNKT	286	IAAMIVNKT	223	MIVNKTVHK	114

Notes: ^1^ Using HLA-DRB1*04 (the closest human homolog to mafa-DRB*w501 of the H1 haplotype of Mauritian Cynomolgus Macaques [98] and the closest human homolog of mafa- DRB1*0402 of the H6 haplotype), HLA-DRB1*09 (the closest human homolog to mafa-DRB1*1001 or mafa-DRB1*1002 of the H2 and H3 haplotype, respectively), HLA-DRB1*16 (the closest human homolog to mafa-DRB4*0101 of the H4 haplotype), and HLA-DRB5*01 (the closest human homolog to mafa-DRB5*0301 of the H5 haplotype), NetPan MHC II web tools were used to predict potential mafa-MHC class II binding peptides. ^2^ Potential mafa-A63 MHC class I binding epitope within the longer peptide sequence shown in bold text. ^3^ Also identified as a possible human linear B cell epitope in Ref. [55]. ^4^ Also identified as a possible human CTL epitope in Ref. [55].

**Table A5:** VaxiJen prediction of microsphere peptide immunogenicity.

ID	Peptide Sequence	VaxiJen Score
SD9	SPDFTNYED	**1.31**
IQ9	IPEPPRQSQ	0.156
HP9	HPAVSSQDP	**1.00**
LQ9	LPPPPLYAQ	**0.872**
TA9	TPVEVTVQA	**1.16**
PL9	PPESLITAL	−0.094
EI9	EPPRQSQDI	**0.482**
LE9	LPPVQEDDE	**0.669**
EP9	EPIRSPSSP	−0.484
YN9	YPNDLLQTN	0.212
KF9	KVKNEVASF	**0.610**
AL9	APFARVLNL	**0.6130**
RV9	RPTALPPPV	**0.9853**
HL9	HYSPLILAL	**1.4600**

Notes: VaxiJen scores > 0.4 predicted to be immunogenic are shown in bold text.

**Table A6:** Unique characteristics of the microsphere vaccine platform relative to recombinant viral vaccines and bare peptide vaccines.

Advantages	Limitations
**Cost-Effectiveness**: Readily synthesized and peptides purified at low cost, making them economically viable. Deployable in areas where ring vaccination strategies might fail. Uses off-the-shelf reagents, simplifying the production process.	**HLA Type Restriction**: Class I MHC restriction limits the relevance of individual peptides to certain HLA types, reducing universality.
**Stability**: Stable at room temperature for more than six months, ensuring a longer shelf-life. Freedom from cold-chain logistic limitations.	**Immune Response**: T cell immune responses may be transient and/or of low magnitude, potentially impacting long-term efficacy.
**Safety**: Synthetic peptides have demonstrated safety in many human studies. The controlled-release adjuvanted microsphere–short peptide vaccine used in this study has demonstrated safety in rodent and NHP models.	**Epitope Diversity**: Peptide vaccine may have to include a large number of epitopes to confer disease protection across a wide range of patients.
**Specificity and Targeted Delivery**: Using defined epitopes avoids uncharacterized antigens that may cause non-therapeutic or autoimmune activity. Microsphere diameter is optimized to target APCs with phagocytic properties and avoids nonprofessional nonphagocytic APCs.	**Induction of B cell antibody responses**: Currently, there is limited information regarding Ebola or Marburg linear B cell epitopes [48] that might be included in the microspheres to induce an effective antibody response. At present, the microsphere platform is a “T cell vaccine” only.
**Monitoring**: Known MHC class I and class II epitope sequences enable direct monitoring of T cell responses, enhancing vaccine efficacy assessment.	**Disease protection vs. Sterilizing immunity**: T cell vaccines may not provide sterilizing immune protection, although they may prevent disease
**Booster Vaccines**: Feasibility of repeated booster vaccines to maintain or enhance immune responses No anti-vector immune response.	**Inaccuracy of T cell epitope prediction methods**: Bioinformatic T cell epitope prediction methods fall short of 100% accuracy in the absence of confirmational studies.
**Protection**: Peptide encapsulation in controlled release PLGA microspheres protects T cell epitopes from extracellular degradation.	
**Adjuvant Efficacy**: Microsphere-encapsulated synthetic adjuvants promote optimal co-stimulation molecule expression by targeted APCs.	
**Mucosal Delivery**: Adjuvanted microspheres are suitable for mucosal surface delivery by inhalation, broadening the application scope.	
**Compliance with Standards**: The Microsphere vaccine platform is animal product-free and conforms to halal standards.	
